# Abnormal inter-hemispheric functional cooperation in blepharospasm

**DOI:** 10.3389/fneur.2025.1660039

**Published:** 2025-10-30

**Authors:** Jian-Ping Liu, Yu-Fang Gu, Yang Shi, Shu-Fang Wang, Chun-Mei Song, Bi-Qin Liu, Ying-Zhu Chen, Hua-Liang Li

**Affiliations:** ^1^Department of Neurology, Affiliated Hospital 6 of Nantong University, Yancheng, China; ^2^Department of Neurology, Yancheng Third People's Hospital, The Affiliated Hospital of Jiangsu Medical College, Yancheng, China; ^3^School of Nursing and Public Health, Yangzhou University, Yangzhou, Jiangsu, China; ^4^Department of Neurology, Yangzhou Wutaishan Hospital of Jiangsu Province, Teaching Hospital of Yangzhou University, Yangzhou, Jiangsu, China; ^5^Department of Neurology, Northern Jiangsu People's Hospital Affiliated to Yangzhou University, Yangzhou, Jiangsu, China; ^6^Department of Geriatrics, Northern Jiangsu People's Hospital Affiliated to Yangzhou University, Yangzhou, Jiangsu, China

**Keywords:** blepharospasm, inter-hemispheric functional cooperation, resting-state functional magnetic resonance imaging, putamen, precentral gyrus

## Abstract

**Background:**

Blepharospasm, characterized by involuntary contractions of the orbicularis oculi muscles, significantly impairs the quality of life. Its pathophysiology remains unclear. Inter-hemispheric cooperation is a prominent feature of the human brain. This study utilizes resting-state functional magnetic resonance imaging (rs-fMRI) to explore inter-hemispheric functional cooperation in blepharospasm patients by examining connectivity between functionally homotopic voxels (CFH), aiming to identify neural disruptions associated with the disorder.

**Methods:**

We recruited 30 patients with blepharospasm and 30 age-, sex-, and education-matched healthy controls. All participants underwent rs-fMRI scanning. CFH maps were generated for each participant to quantify inter-hemispheric connectivity at the voxel level. Group differences were assessed, and partial correlation analyses were performed in the patient group to examine the relationship between aberrant CFH values and clinical variables.

**Results:**

Compared to healthy controls, patients with blepharospasm showed significantly increased CFH in the left putamen and left precentral gyrus. However, these aberrant CFH values did not significantly correlate with clinical variables, including disease duration or total Jankovic Rating Scale (JRS) scores and its subscales.

**Conclusions:**

This study identifies increased inter-hemispheric functional connectivity (FC) within key motor-related brain regions in blepharospasm. The observed hyperconnectivity in the putamen and precentral gyrus may reflect a compensatory neural mechanism to counteract motor dysfunction. These findings provide novel insights into the pathophysiology of blepharospasm and suggest that modulating inter-hemispheric communication may be a potential therapeutic target.

## 1 Introduction

Blepharospasm is a focal dystonia characterized by involuntary, repetitive contractions of the orbicularis oculi muscles, resulting in excessive blinking and forced eyelid closure. Although its precise etiology remains elusive, both genetic and environmental factors are believed to contribute to its development, with dysfunction in the basal ganglia and cortical regions implicated in its pathophysiology (1). Blepharospasm can severely impact quality of life, leading to functional blindness in advanced cases, underscoring the need for a deeper understanding of its neural mechanisms to develop effective treatments (2, 3).

The advancement of neuroimaging techniques has provided new opportunities for investigating the neural mechanisms underlying dystonic disorders. Resting-state functional magnetic resonance imaging (rs-fMRI), in particular, has emerged as a valuable tool for exploring the intrinsic functional architecture of the brain (4). By examining spontaneous brain activity, rs-fMRI allows for the assessment of functional connectivity (FC) between different brain regions, offering insights into the complex network dynamics that may be disrupted in neurological disorders. One novel approach to understanding these disruptions is the analysis of connectivity between functionally homotopic voxels (CFH) (5). CFH quantifies inter-hemispheric cooperation by measuring the functional connectivity between corresponding regions in the left and right hemispheres, thus offering a nuanced perspective on hemispheric communication essential for integrated brain function (5). Unlike voxel-mirrored homotopic connectivity (VMHC), which presumes anatomical symmetry and examines correlations between each voxel and its mirrored counterpart in the opposite hemisphere (6), CFH directly reflects genuine functional correspondence, unconstrained by perfect anatomical symmetry. Abnormal CFH has been reported in various neurological and psychiatric conditions, including Parkinson's disease (5), Alzheimer's disease (7), schizophrenia (8), and major depressive disorder (9), suggesting that impaired inter-hemispheric communication may be a common feature of these disorders. Previous studies have demonstrated aberrant VMHC in multiple brain regions—including the inferior temporal gyrus, inferior frontal gyrus, posterior cingulate cortex, and postcentral gyrus—in patients with blepharospasm compared to healthy controls (10), as well as in other forms of dystonia (11, 12). This evidence suggests that impaired inter-hemispheric coordination is a key feature of the disorder, making it plausible that disruptions in the related measure of CFH could play a significant role in its pathophysiology.

Accordingly, the aim of the present study was to examine CFH in patients with blepharospasm using rs-fMRI. We hypothesized that these patients would display significant CFH abnormalities, particularly in regions involved in motor control and sensory processing. By identifying specific patterns of inter-hemispheric connectivity disruption, this research seeks to advance our understanding of the neural mechanisms underlying blepharospasm and foster the development of targeted therapeutic interventions.

## 2 Methods

### 2.1 Participants

This study recruited 30 patients with primary blepharospasm (13), a condition characterized by involuntary eyelid closure due to spasms in the orbicularis oculi muscles, as well as increased blinking or a sensory trick. Inclusion for the patient group was based on established diagnostic criteria for primary blepharospasm (13) and did not have a history of neurological, major psychiatric, or ophthalmologic diseases that could secondarily cause eyelid spasms (e.g., severe dry eye syndrome, blepharitis, trichiasis, or structural eyelid abnormalities), alcohol or drug abuse, structural lesions on MRI, or a family history of movement disorders. To avoid the confounding effects of treatment, patients were newly diagnosed and treatment-naïve, having never received botulinum toxin injections or other specific therapies for blepharospasm at the time of enrollment or during the MRI scans. Thirty healthy controls, matched for age and sex, were recruited from the local community via advertisements. None of the participants had a history of neuropsychiatric or medical illness, and there were no family histories of psychiatric or neurological disorders. All participants were right-handed. Demographic information and clinical data were gathered for all individuals.

Before the rs-fMRI examination, the severity of blepharospasm was evaluated using the Jankovic Rating Scale (JRS) (14), which includes subscales for measuring the severity and frequency of eyelid spasms. Additionally, all participants were assessed for depressive and anxiety symptoms using the Self-Rating Depression Scale (SDS) (15) and the Self-Rating Anxiety Scale (SAS) (16).

All subjects provided written informed consent, and the study protocol was approved by the Research Ethics Review Board of Yancheng Third People's Hospital.

### 2.2 Rs-fMRI data acquisition

Rs-fMRI data were acquired on a 3-Tesla scanner (Discovery 750; GE Healthcare, Milwaukee, WI, USA). Foam padding and headphones were used to minimize head motion and reduce scanner noise. Participants were instructed to keep their eyes closed and stay awake during the scanning sessions. Functional images were acquired using a gradient-recalled echo-planar imaging (GRE-EPI) with the following parameters: repetition time (TR) = 2,000 ms, echo time (TE) = 30 ms, flip angle = 90°, field of view = 240 × 240 mm^2^, matrix = 64 × 64, slice number = 35, slice thickness = 4 mm, no slice gap, NEX = 1, voxel size = 3.75 × 3.75 × 4 mm^3^, and a total of 230 time points.

### 2.3 Preprocessing of rs-fMRI data

Functional data were preprocessed using the Data Processing and Analysis of Brain Imaging (DPABI) toolbox (17) (http://rfmri.org/dpabi), a toolbox based on Statistical Parametric Mapping (SPM12, http://www.fil.ion.ucl.ac.uk/spm) and implemented in MATLAB (version R2018b, The MathWorks, Natick, MA, USA).

To ensure stable longitudinal magnetization, the first 10 volumes were removed. The preprocessing steps included adjusting for differences in acquisition time between slices (slice timing correction), correcting for head motion by aligning all functional images to the first image in the series (realignment), transforming images to the Montreal Neurological Institute (MNI) space and segmenting into gray matter, white matter, and cerebrospinal fluid (normalization), applying a 4-mm full-width at half-maximum (FWHM) isotropic Gaussian kernel for smoothing, regressing out 24 head-motion parameters, mean signals in the whole brain, white matter, and cerebrospinal fluid for nuisance regression, and filtering the time series to retain frequencies between 0.01 and 0.1 Hz (band-pass filtering).

### 2.4 Calculation of CFH

In line with a growing body of literature (5, 8, 9), we employed the CFH method to assess inter-hemispheric cooperation. It is crucial to distinguish the methodological definition of this term from its potential statistical outcomes. “Functional homotopy” here refers to the data-driven identification of the most highly correlated voxel pair between functionally corresponding contralateral regions, making it a strictly homotopic measure. A key strength of this approach, unlike methods assuming perfect anatomical symmetry, is its ability to reveal spatially asymmetric or lateralized group differences, which may reflect the underlying functional specialization and pathology of the brain.

CFH analysis was performed to evaluate the connectivity between functionally homotopic regions across hemispheres, following the methodology described in previous studies (5, 8, 9). This data-driven process involved two main steps. First, for each voxel in one cerebral hemisphere, its FC with every voxel in the contralateral hemisphere was computed. The voxel in the opposite hemisphere that exhibited the maximal FC was identified as its functionally homotopic counterpart. Second, the CFH value was defined as the Pearson's correlation coefficient between the BOLD time series of the seed voxel and its identified functionally homotopic voxel. It is important to note that this “functionally homotopic” counterpart is defined by the maximal functional correlation, rather than by strict anatomical mirroring, thereby reflecting genuine functional correspondence. This data-driven approach distinguishes CFH from methods like VMHC, which presumes perfect anatomical symmetry. To improve normality for statistical analysis, all correlation coefficients were converted to Fisher's *z*-scores, generating a whole-brain CFH map for each participant. These CFH values, which are not constrained by strict anatomical symmetry, provide a voxel-wise index of intrinsic inter-hemispheric functional integration. Group-level analyses were subsequently performed using these individual CFH maps.

### 2.5 Statistical analysis

The demographic and clinical characteristics of patients with blepharospasm were compared to those of healthy controls using IBM SPSS Statistics 22.0 software (IBM Corp., Armonk, NY, USA), utilizing the Chi-square test, two-sample *t*-test, or Mann–Whitney *U* test as appropriate, based on data distribution. A *p*-value < 0.05 was considered statistically significant.

A group comparison of CFH maps between patients with blepharospasm and healthy controls was performed using SPM12 within a gray matter mask (probability threshold >0.2). A permutation test in the Statistic Non-Parametric Mapping (SnPM13) toolbox of SPM12 was used for statistical analysis with age, sex, education, SDS, and SAS as covariates. A voxel-level threshold of *p* < 0.001 was applied, along with a cluster level threshold set at *p*_corr_ < 0.05.

### 2.6 Correlation with clinical variables

In regions where significant group differences in CFH were observed, the values were extracted and then correlated with clinical variables such as disease duration and JRS, along with its subscales, using partial correlation analyses while controlling for age, sex, education, SDS, and SAS. A value of *p* less than 0.05 was deemed statistically significant.

## 3 Results

### 3.1 Demographic and clinical characteristics

The demographic and clinical characteristics of the participants are summarized in Table 1. There were no significant differences between patients with blepharospasm and healthy controls in terms of age, sex, or education level (*p* > 0.05 for all). The mean illness duration for blepharospasm patients was 12.33 ± 4.9 months. The median total JRS score among blepharospasm patients was 3 (range: 2–6). Compared to healthy controls, blepharospasm patients exhibited significantly higher anxiety levels (SAS: 43.33 ± 6.73 vs. 36.67 ± 5.44; *p* < 0.005). Similarly, depression levels were also elevated in the patient group (SDS: 44.97 ± 6.45 vs. 38.40 ± 5.26; *p* < 0.005).

**Table 1:** Demographic and clinical characteristics of participants.

**Characteristics**	**Blepharospasm (*N* = 30)**	**Controls (*N* = 30)**	***p-*value**
Age (years)^†^	51.07 (6.46)	49.93 (5.99)	0.48
Gender (M/F)	12/18	12/18	1.0
Education (years)^†^	9.40 (3.62)	9.97 (3.96)	0.57
Illness duration (months)^†^	12.33 (4.9)		
JRS_total score^‡^	3 (2–6)	–	–
JRS_severity^‡^	1 (1–3)	–	–
JRS_frequency^‡^	2 (1–3)	–	–
SAS score^†^	43.33 (6.73)	36.67 (5.44)	<0.005
SDS score^†^	44.97 (6.45)	38.40 (5.26)	<0.005

M, male; F, female; JRS, Jankovic Rating Scale; SAS, Self-rating Anxiety Scale; SDS, Self-rating Depression Scale.

^†^ Data presented as mean (standard deviation).

^‡^ Data presented as median (range).

### 3.2 Between-group differences in CFH

**Figure 1:**
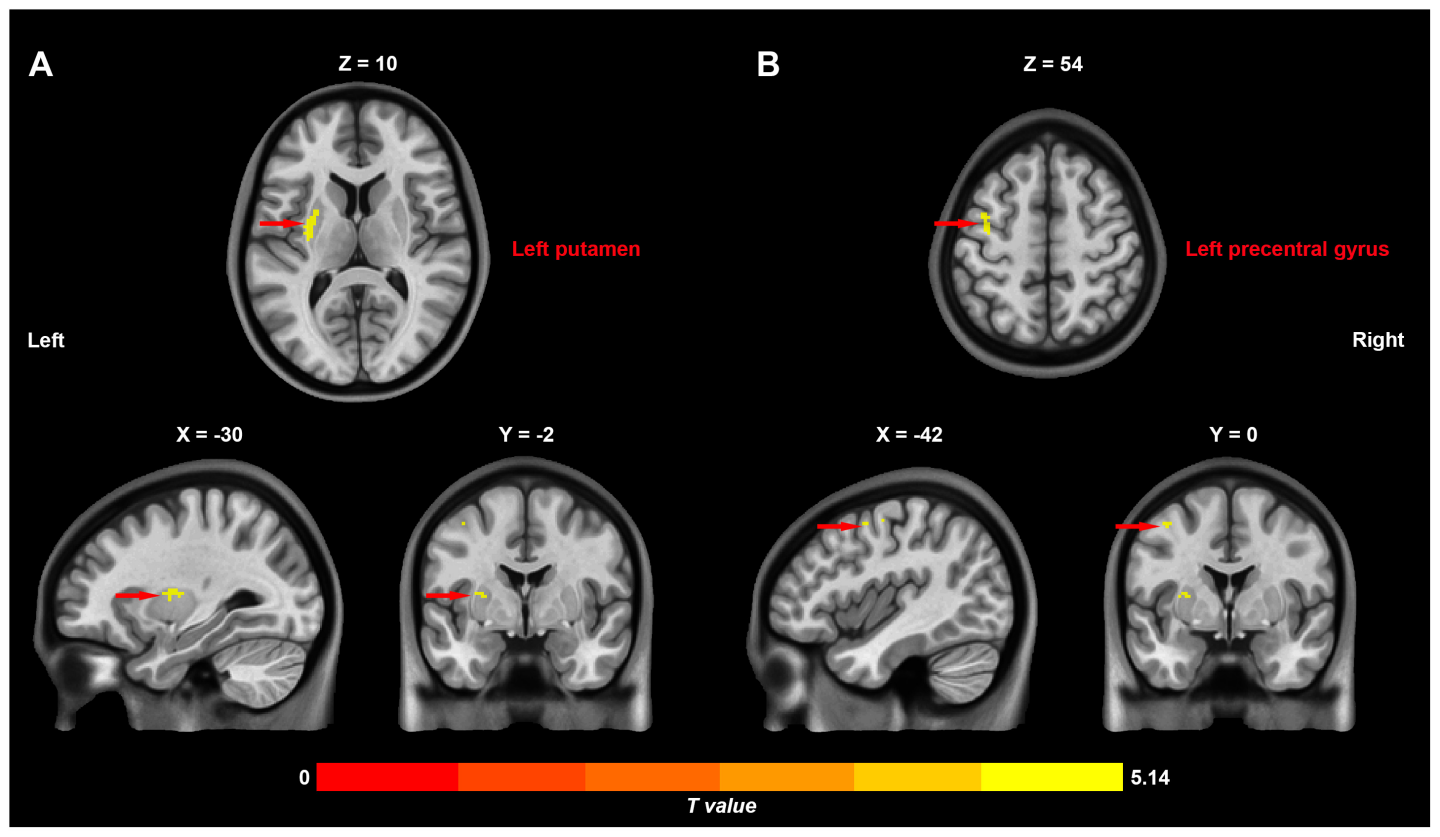
Differences in inter-hemispheric cooperation between patients with primary blepharospasm and healthy controls. Increased connectivity between functionally homotopic voxels in the left putamen **(A)** and left precentral gyrus **(B)** in patients with primary blepharospasm compared to healthy controls.

As shown in Figure 1, rs-fMRI analysis revealed significant differences in CFH between patients with blepharospasm and healthy controls. Specifically, patients with blepharospasm exhibited increased CFH in the left putamen (peak *t*-value = 5.14, MNI coordinates = [−30, −2, 10], cluster size = 53 voxels, *p*_corr_ < 0.05) and left precentral gyrus (peak *t*-value = 4.98, MNI coordinates = [−42, 0, 54], cluster size = 27 voxels, *p*_corr_ < 0.05) compared to healthy controls after controlling for age, sex, education, SAS, and SDS.

### 3.3 Correlation with clinical variables

In the patient group, partial correlation analyses were conducted controlling for age, sex, education, SAS, and SDS. These analyses revealed no significant correlations between the aberrant CFH values in either the left putamen or the left precentral gyrus and disease duration or JRS scores (total and subscales; *p* > 0.05 for all).

## 4 Discussion

Our study identified significant alterations in CFH among patients with blepharospasm, specifically demonstrating increased CFH in the putamen and precentral gyrus. These results provide important insights into the neural mechanisms of blepharospasm.

The putamen, a crucial component of the basal ganglia, plays a key role in regulating motor control and executing voluntary movements (18, 19). The observed increase in CFH within the putamen indicates heightened inter-hemispheric coordination in this region among blepharospasm patients. While direct anatomical connections between the putamina are sparse, this functional connectivity likely reflects synchronized activity mediated through polysynaptic cortico-striatal-thalamo-cortical loops, which are coordinated between the two hemispheres. This may represent a compensatory process by which the brain attempts to counteract impaired motor control. The putamen may exert greater effort to integrate motor signals and fine-tune movement patterns, thereby supporting normal function in response to the frequent and involuntary muscle contractions characteristic of blepharospasm. Previous research has implicated the basal ganglia, particularly the putamen, in the pathophysiology of dystonic disorders (18, 20, 21). Abnormalities in this region have been associated with impaired inhibition and altered sensory-motor integration, which are key features of blepharospasm. The observed increase in CFH may reflect an adaptive response aimed at enhancing inter-hemispheric communication to mitigate these impairments. This aligns with studies in other dystonic disorders where increased connectivity within the basal ganglia network has been reported as a compensatory mechanism to preserve motor function (21).

The precentral gyrus, also known as the primary motor cortex, plays a pivotal role in the planning and execution of voluntary movements. Increased CFH in the precentral gyrus indicates enhanced inter-hemispheric coordination in the primary motor cortex of blepharospasm patients. This finding is particularly relevant given the precentral gyrus's involvement in generating motor commands and its influence on muscle activity. Enhanced CFH in the precentral gyrus may suggest an increased need for bilateral motor cortex communication to control the excessive and involuntary muscle contractions seen in blepharospasm (2). This could be interpreted as an attempt by the brain to synchronize motor outputs and achieve more coordinated and controlled muscle movements, despite the underlying dystonic disruptions. The primary motor cortex's involvement in both initiating and modulating motor activity underscores the importance of inter-hemispheric connectivity in maintaining motor function in the face of neural abnormalities. Recent research has provided further evidence supporting the idea that the primary motor cortex plays a key role in the development of blepharospasm. Luo et al. (22) discovered abnormal dynamic brain activity and functional connectivity of primary motor cortex in blepharospasm using rs-fMRI (22). Huang et al. (23) also identified notable functional disruptions in the sensorimotor network, including the primary motor cortex (23). Additionally, a multimodal meta-analysis of voxel-based morphometry and functional neuroimaging studies revealed conjoint anatomic and functional changes in left precentral gyrus in idiopathic blepharospasm (24). These studies collectively underscore the critical involvement of the primary motor cortex in the pathophysiology of blepharospasm, with significant abnormalities in functional activity and connectivity within this specific brain region.

Our findings of increased CFH in the putamen and precentral gyrus differ from some previous studies using VMHC, which reported aberrant connectivity in other regions such as the temporal and frontal gyri (9). This discrepancy may be attributable to several factors. First, the CFH and VMHC methods capture different aspects of inter-hemispheric connectivity; CFH is data-driven and reflects functional correspondence, while VMHC is constrained by anatomical symmetry. Second, our cohort consisted of newly diagnosed, treatment-naïve patients, which may present a different neurophysiological profile compared to cohorts with longer disease duration or treatment history. Finally, differences in imaging parameters, such as spatial resolution, and statistical thresholds could also contribute to the observed variations. Future multi-modal studies are needed to reconcile these findings.

Notably, the observed hyperconnectivity was localized to the left hemisphere. As all participants in our study were right-handed, this finding may reflect alterations specific to the dominant motor hemisphere. The left precentral gyrus and its connected basal ganglia circuits are critical for motor control in right-handers, and the increased inter-hemispheric coordination may represent a compensatory effort originating from these key motor hubs. However, this lateralization requires further investigation in future studies.

The findings of increased CFH in both the putamen and precentral gyrus suggest potential targets for therapeutic interventions. Strategies that modulate inter-hemispheric communication or connectivity in these regions could prove beneficial in managing blepharospasm symptoms. For instance, non-invasive brain stimulation techniques like transcranial magnetic stimulation or transcranial direct current stimulation may be used to improve motor control by modulating functional connectivity (25–27). Moreover, gaining insight into the compensatory mechanisms in blepharospasm may help in the creation of interventions that support these adaptive processes. By developing therapies that boost the brain's innate compensatory strategies, there is potential for better management of blepharospasm, ultimately enhancing patients' quality of life and functional outcomes.

### 4.1 Limitations

Although our study provides valuable insights, certain limitations should be acknowledged. First, due to the cross-sectional design, we cannot establish causality regarding the observed alterations in CFH. Longitudinal research is needed to determine whether increased CFH in the putamen and precentral gyrus represents a stable trait or a dynamic response to disease progression. Second, the spatial resolution and number of time points of our fMRI data were modest by current standards. While sufficient to detect group differences in larger structures like the putamen and precentral gyrus, future studies using higher-resolution imaging and longer scan durations could provide more detailed insights and improve the sensitivity for detecting changes in smaller brain nuclei. Third, this study focused exclusively on functionally homotopic connectivity. Future research should also investigate heterotopic (non-homotopic) inter-hemispheric connections to provide a more comprehensive understanding of the complex cross-hemispheric network disruptions in blepharospasm.

## 5 Conclusions

In summary, our identification of increased CFH in the putamen and precentral gyrus provides fresh insights into the neural mechanisms underlying blepharospasm. These results stress the importance of inter-hemispheric communication in addressing motor dysfunction and suggest potential paths for therapeutic intervention. Further investigation is necessary to explore the clinical significance of these findings and to develop strategies that leverage the brain's compensatory mechanisms to improve outcomes for patients with blepharospasm.

## Data Availability

The raw data supporting the conclusions of this article will be made available by the authors, without undue reservation.
